# The Halogen-Bond Nature in Noble Gas–Dihalogen Complexes from Scattering Experiments and Ab Initio Calculations

**DOI:** 10.3390/molecules24234274

**Published:** 2019-11-23

**Authors:** Francesca Nunzi, Benedetta Di Erasmo, Francesco Tarantelli, David Cappelletti, Fernando Pirani

**Affiliations:** 1 Dipartimento di Chimica, Biologia e Biotecnologie, via Elce di Sotto 8, I-06123 Perugia, Italy; benedetta.dierasmo@studenti.unipg.it (B.D.E.); francesco.tarantelli@unipg.it (F.T.); david.cappelletti@unipg.it (D.C.); 2 Istituto CNR di Scienze e Tecnologie Chimiche “Giulio Natta” (CNR-SCITEC), via Elce di Sotto, I-06123 Perugia, Italy

**Keywords:** cross sections, molecular beam scattering, charge transfer, coupled cluster, excited states, ionization potential, electron affinity, interaction potential, stereoselectivity

## Abstract

In order to clarify the nature of the halogen bond (XB), we considered the prototype noble gas–dihalogen molecule (Ng–X_2_) systems, focusing on the nature, range, and strength of the interaction. We exploited data gained from molecular beam scattering experiments with the measure of interference effects to obtain a suitable formulation of the interaction potential, with the support of high-level ab initio calculations, and charge displacement analysis. The essential interaction components involved in the Ng–X_2_ adducts were characterized, pointing at their critical balance in the definition of the XB. Particular emphasis is devoted to the energy stability of the orientational Ng–X_2_ isomers, the barrier for the X_2_ hindered rotation, and the influence of the X_2_ electronic state. The present integrated study returns reliable force fields for molecular dynamic simulations in Ng–X_2_ complexes that can be extended to systems with increasing complexity and whose properties depend on the selective formation of XB.

## 1. Introduction

The intermolecular halogen bond (XB), the non-covalent interaction involving an electrophilic region of a halogen atom, is known to control important static and dynamic properties of matter [1]. The role and selectivity of the XB are of high relevance in supramolecular chemistry for the synthesis of materials with a wide variety of technological properties and drug design [2,3]. Moreover, there are important energy transfer processes and predissociation phenomena where the chemical kinetics is guided by interaction forces associated with the XB formation [4,5,6,7,8].

Intermolecular interactions are usually regarded as constituted by two kinds of main contributions: electrostatic (*V_el_*) and non-electrostatic (*V_nel_*) [9,10]. The former contribution originates from the interaction of charges and permanent electric dipoles or multipoles present in the interacting species. *V_el_* is often dominating and it makes the extent and role of *V_nel_*, although often important, difficult to quantify. The nature of *V_nel_* is difficult to assess precisely, as it arises from the delicate balance of various components: Pauli size-repulsion (*V_sr_*), dispersion (*V_d_*), and induction (*V_i_*) attractions, and charge-transfer (*V_ct_*), which all possess different dependence and scaling properties with the distance and relative orientation of the interacting partners. In this respect, the study of simple systems with high orientational symmetry, and where *V_el_* is small or even vanishing, is especially important to understand and model the various *V_nel_* components. Noble-gas–halogen adducts, Ng–X_2_ (X = Cl, Br, I), are such ideal prototypes to investigate the role of *V_nel_* components in XB emergence. In a recent study we carried out, the Ng–Cl_2_ system showed, for example, that XB formation selectively stabilizes the collinear configuration of the adducts when Cl_2_ is in its *X* ^1^*Σ_g_* ground state [11], while this effect disappears if Cl_2_ is in its first triplet excited state, *B* ^3^*Π_u_* [12,13]. We further studied in detail how the XB formation and the charge transfer (CT) component make the interaction potential deviate from the typical van der Waals model, *V_vdW_* (defined as *V_vdW_* = *V_sr_* + *V_d_*), and how these additional components can be accurately modeled in general [12].

In the present work, we build on our previous findings in order to characterize the entire family of Ng–X_2_ complexes. This systematic study will enable us to establish, in a consistent fashion, trends and features that govern the XB formation, and its dependence on the nature of Ng and X_2_, in an effort to continue the development of a general predictive model to describe the XB—in particular, components other than the electrostatic one, which is of crucial importance for the design and analysis of more complex systems. As in previous works [13,14,15,16], we use an integrated phenomenological–theoretical approach. The phenomenological procedure is based on the available data resulting from collisional experiments, combined with (semi)empirical methods developed over the years to predict the range and strength of weak interactions and to provide an adequate analytical representation of the potential energy surface (PES). This is flanked by highly accurate theoretical ab initio calculations, which allow us to validate and improve the PES and the models, and to understand and characterize the fundamental components of the interaction. Key to this approach is our ab initio charge–displacement (CD) analysis, which enables us in particular to assess and quantify in a transparent and consistent way the elusive CT component [16].

## 2. Results and Discussion

### 2.1. Scattering Results

**Figure 1:**
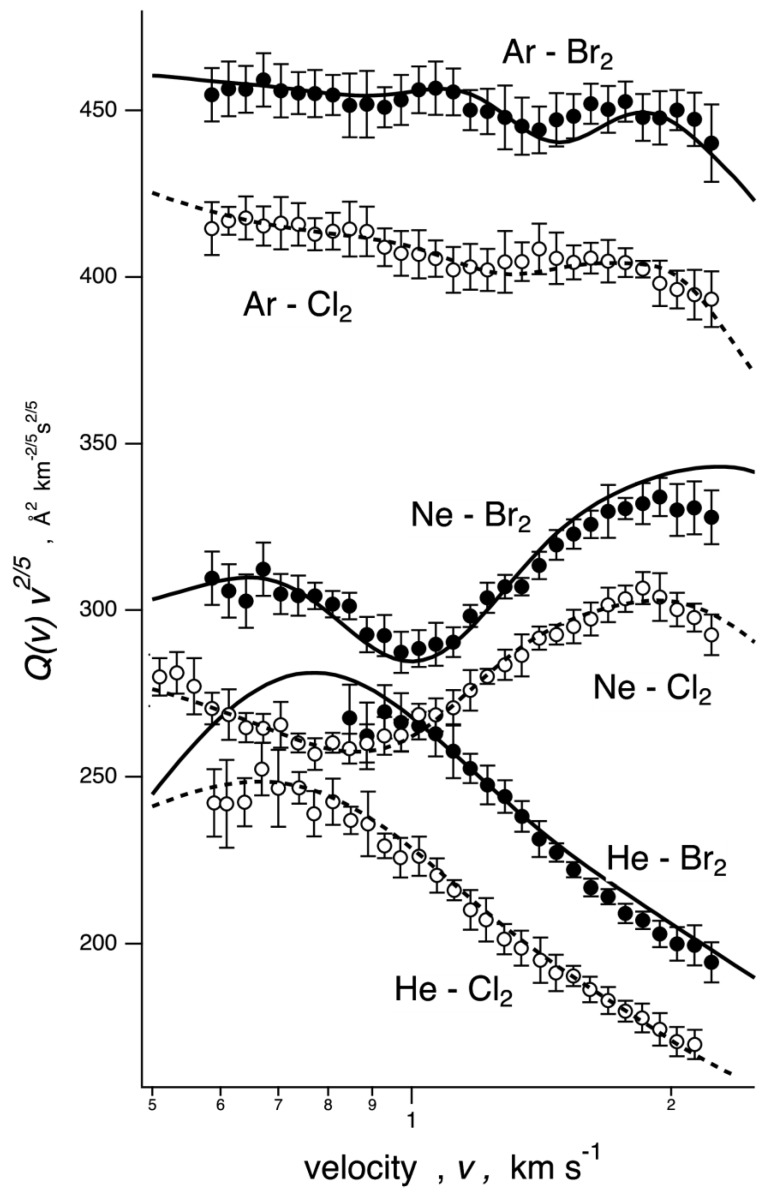
Integral cross sections *Q*(*v*) for noble gas (Ng) atom projectiles colliding at each selector velocity *v* with Cl_2_ and Br_2_ targets. The black solid lines are the best-fit cross section calculated from the model potential (see text); the dashed lines refer to the spherically component of the potential energy surfaces (PESs).

The total integral cross section *Q*(*v*), as a function of the collision velocity *v*, was measured by means of the molecular beam technique, the noble gases He, Ne, Ar being the projectiles, and the electronic ground state Cl_2_/Br_2_ halogen molecules being the targets [12,13,17]. The results are reported in Figure 1 and clearly point out that the measured cross-section is made up of the superposition of a smooth average component and an oscillating pattern arising from the quantum “glory” interference. In particular, the cross sections were plotted as *Q*(*v*)·*v*^2/5^ to emphasize the well resolved oscillatory patterns due to the quantum interference. As is well known [18,19], the average component is directly related to the absolute scale of the long range mean attractive interaction potential of the colliding system. Frequency, amplitude, and position of the ”glory” oscillations are instead determined by the absolute value, form, and anisotropy of the potential near the equilibrium configuration. Overall, the collected scattering data returned direct information on the basic features of the phenomenological PES describing the intermolecular interaction in Ng–X_2_ systems. The data in Figure 1 are evidence that both the smooth average and the oscillating cross section components vary along the Ng series for a selected halogen molecule and with the halogen moiety for a selected Ng, attesting to the sensitivity of the experiment to the details of the interaction.

### 2.2. Interaction Potential Components

An analytical form of the interaction PES, which in this case is expressed as a function of the vector **R** length, with **R** joining Ng and X_2_ midpoint, and of the *Θ* angle between **R** and X_2_ axis (see Figure S1 in the Supplementary Materials, SM), was used to fit the observable experiment by tuning a few physically meaningful parameters defining strength and range of the fundamental interaction components. Specifically, each PES was defined as a combination of the ubiquitous van der Waals (*V_vdW_*) component, given in a suitable anisotropic form, with a three-body contribution *V*_3*B*_, arising from the strongly asymmetric electronic charge distribution around the X_2_ molecule, to which was added the stereo-selective role of a CT stabilization effect (*V_CT_*). An important test of the analytical form of the interaction potential and of its parametrization was obtained by calculating the scattering cross sections for each system from the spherical average and from the full PESs, to reproduce, at low and high collision velocity, respectively, values and velocity dependence of the measured data, including also the frequency and amplitude of the oscillating patterns. In particular, results calculated under the two limiting conditions were combined according to the different dynamical regimes, emerging in different collision velocity ranges (see refs [12,13,17] and below), in order to provide a better comparison with the experimental data. As it is shown in Figure 1, the employed potential model adequately reproduced cross section values and both amplitude and frequency of the glory patterns observed in the investigated Ng–X_2_ systems. The potential analytical form was further validated by comparing selected cuts of the phenomenological PESs with the ab initio ones (see the next sections).

When experimental cross section data are missing, the analytical expression for the PES can be gained by merely adopting potential parameters, obtained by proper scaling laws defined in terms of fundamental physical properties of the interacting partners [10,11,12,13,14], and then applying the fine-tuned phenomenological model potential—see the Ng–X_2_ complexes involving the heavier (Kr, Xe) Ngs or I_2_ and/or when X_2_ is in the lowest excited *B* state.

**Figure 2:**
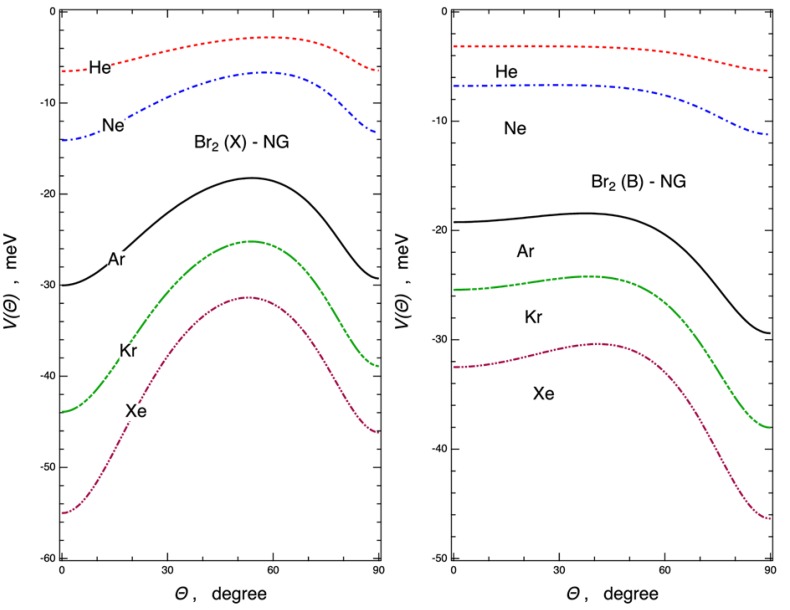
Angular minimum energy path (MEP) for the Ng–Br_2_ complexes in the ground *X* ^1^*Σ_g_* (left) and excited *B* ^3^*Π_u_* (right) state of Br_2_, reporting the total interaction energy *V*, as derived from the potential parametrization, vs the angular variable *Θ*.

**Figure 3:**
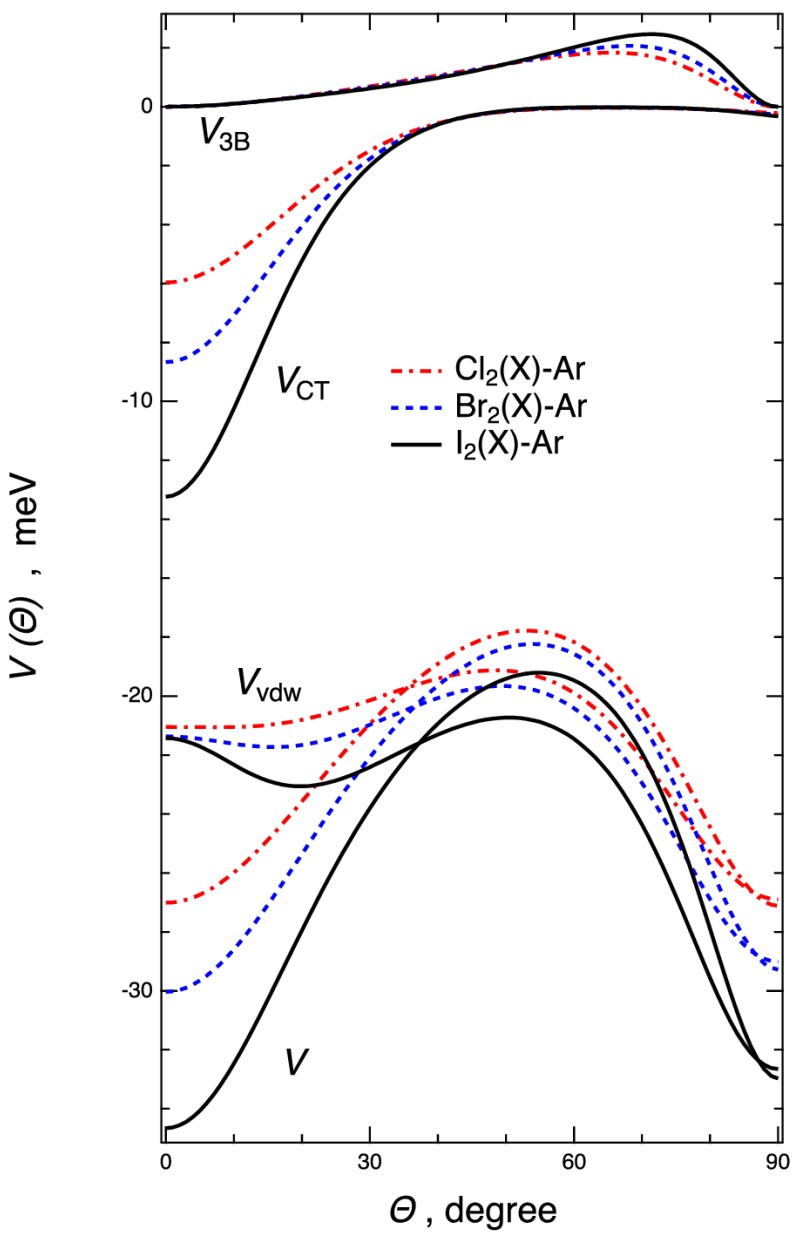
Angular MEP for the Ar–X_2_ complexes in the ground *X* ^1^*Σ_g_* state of X_2_ (X = Cl, Br, I), reporting the overall interaction energy *V* and its components (*V_vdw_*, *V_CT_*, and *V*_3*B*_), as derived from the potential parametrization, vs the angular variable, *Θ*.

Further details on the PES parametrization are given in the next section. The main features of the obtained PESs, in terms of equilibrium distances *R_m_* and binding energies *E_m_* are given in Table 1 and Table 2 for a useful comparison of all systems. Since general and common trends were observed among the Ng–X_2_ complexes, selected values related to Ng–Br_2_ and Ar–X_2_ (X = Cl, Br, I) complexes were considered, as seen in Figure 2 and Figure 3, showing the angular minimum energy path (MEP) associated with the PESs (see Figures S2–S5 in SM for Ng–Cl_2_ and Ng–I_2_ systems). For the ground state Ng–Br_2_(*X*) complexes, the Figure 2 (left side) emphasizes the increase of the interaction along the Ng series and shows the occurrence of a saddle point at intermediate orientation angle (*Θ* is about 50–60°). For each system the ground state linear configuration (*Θ* = 0°) results were more stable than those of the excited state (right side of Figure 2) and for heavier Ng–Br_2_(*X*) systems, such configuration became in absolute the most stable one. In all cases a relevant selectivity arose from the different angular dependence of the interaction components involved. The various components concurring with the overall interaction potential, evaluated along the MEPs, are reported in Figure 3 for the selected Ar–X_2_(*X*) adducts (X = Cl, Br, I). Interestingly, it points out that the *V_vdW_* component tended to stabilize the perpendicular configuration (*Θ* = 90°), while the *V_CT_* contribution emerged more efficiently in proximity of the collinear configuration, and *V*_3*B*_ provided a less important, although repulsive, contribution at intermediate configurations. We verified that for the excited PESs the contributions of *V*_3*B*_ and *V_CT_* were found to be vanishing, suggesting that the involved interactions showed a pure van der Waals behavior. Therefore, the full topography of each PES depended on the critical balance of the effectiveness of the *V_vdW_*, *V_CT_*, and *V*_3*B*_ components. All these peculiarities of the intermolecular interaction in Ng–X_2_ complexes were confirmed by the ab initio calculations (see below).

### 2.3. Ab Initio Potential Energy Surfaces

**Figure 4:**
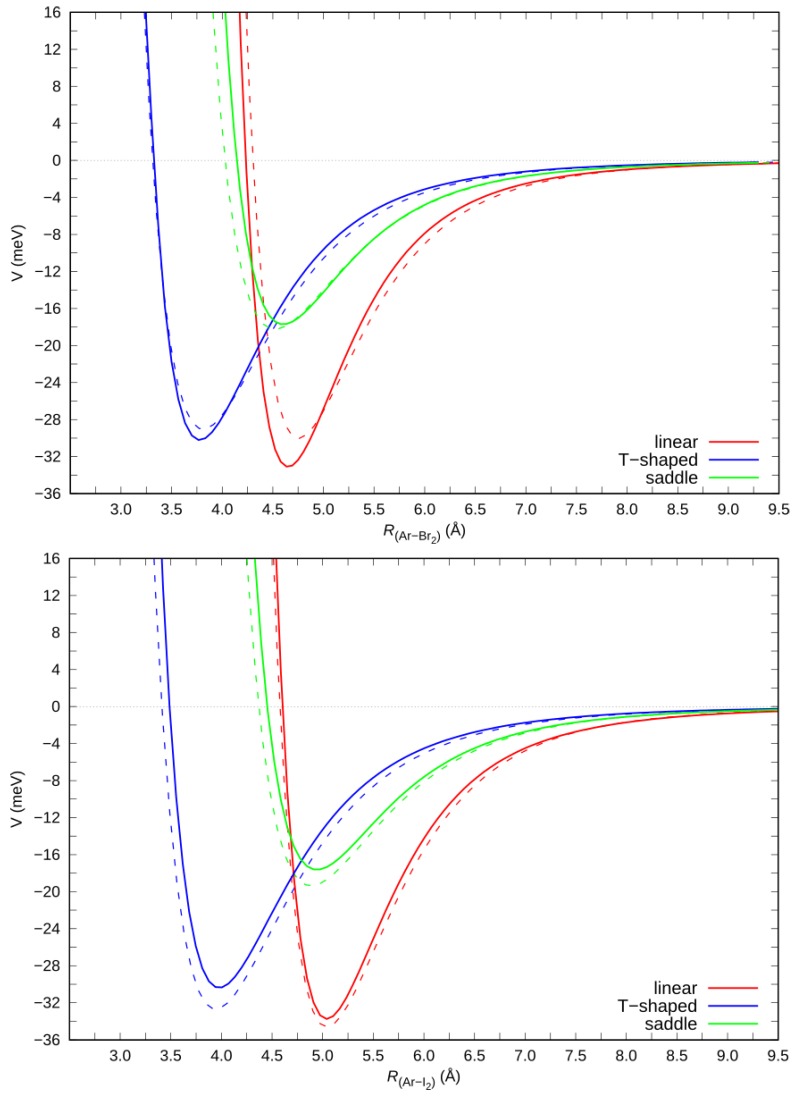
Solid lines: potential energy curves (interaction potential *V* vs Ar–X_2_ distance *R*) for Ar–Br_2_ (top, CCSD(T)/AV5Z) and Ar–I_2_ (bottom, CCSD(T)/AVQZ) in their ground state. Dashed lines: corresponding curves obtained from the semi-empirical model.

As discussed above, ab initio calculations were performed on the Ng–X_2_ adducts aimed at assessing the leading interaction components, with a particular focus on their origin in the electron density displacement, and to further test the validity of the adopted intermolecular potential formulation. We focused on the cuts of the PESs most relevant for the anisotropic behavior of the Ng–X_2_ interaction, which are the linear and the T-shaped configurations. For the saddle isomer both the ab initio and the phenomenological PESs return a *Θ* value of ca. 50–60° for all the Ng–X_2_ systems. The ab initio computed cuts of the PES for the three considered configurations were compared with those derived from the semi-empirical potential model parametrization, eventually fitted through the analysis of cross section data. In Figure 4 we report the ab initio and model cuts of the PES for the Ar–X_2_ complexes (X = Br, I) in the *X* ground state, while those for the Cl_2_ adducts are reported in Reference [13]. A satisfactory agreement (within the combined own uncertainty) was clearly indicated between the ab initio and the model potentials, thus further confirming the validity of the parametrized semi-empirical model for the accurate representation of the Ng–X_2_ interaction (X = Cl, Br, I) over the whole range of interaction distances, orientation angles, and strengths. The optimized intermolecular distance (*R_m_*) of the equilibrium structures and the corresponding binding energies (*E_m_*) of the considered cuts are reported in the SM section, Table S1.

**Figure 5:**
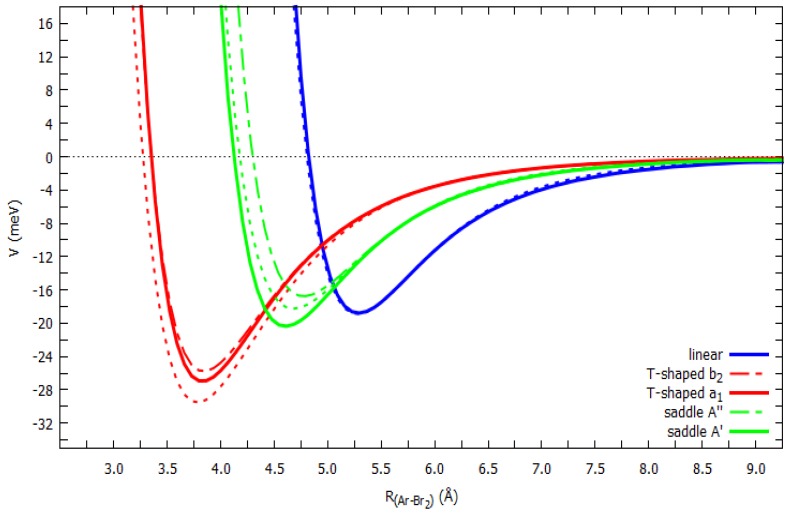
Potential energy curves (interaction potential *V* vs Ar–Br_2_ distance *R*) for the Ar–Br_2_ system, with the Br_2_ moiety in the (*B* ^3^*Π_u_*) excited state, computed at CCSD(T)/AV5Z level of theory (solid and dotted–dashed lines) and calculated with the semi-empirical method (dotted lines).

For the selected Ar–Br_2_ adduct, we also report in Figure 5 the computed cuts of the electronically excited PES, compared with those provided by the phenomenological potential model. Here too, the agreement was entirely satisfactory. The interpretation afforded by the model revealed that, at variance with the ground state, the interaction in the excited state was entirely of van der Waals nature, thus explaining the higher energy stability of the T-shaped vs linear configuration, with the *V_CT_* and *V*_3*B*_ components playing a minor role.

### 2.4. Halogen Polar Flattening in the Ng–X_2_ Complexes

**Figure 6:**
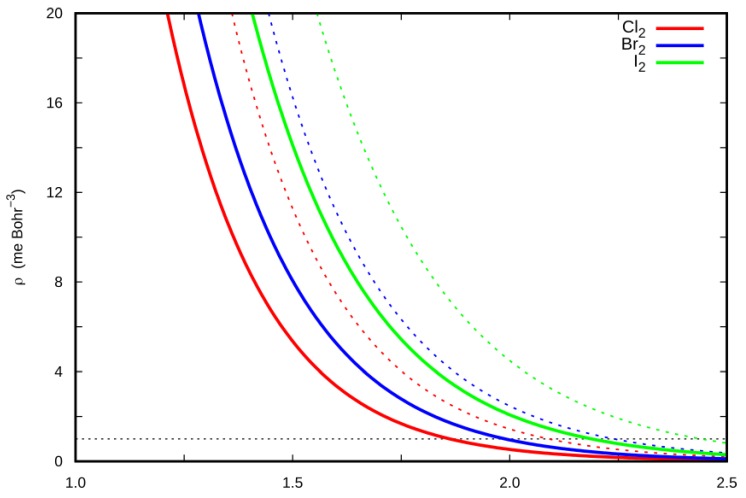
X_2_ electron density profile, *ρ* (me Bohr^−3^), on the outer side of the X_2_ molecule along the X_2_ bond (solid lines) and perpendicular to it at the X nucleus (dotted lines).

A fundamental enabling feature of the XB is the so-called σ-hole on the halogen atoms, the axial electron deficiency that permits an approaching donor to bind. Associated with the σ-hole is a flattening of the halogen electron density on the outer side of the X_2_ molecule along the bond direction. Remarkably, the strong anisotropy of the halogen electron density in the X_2_ molecule can be related to a shift at lower distances of the repulsive wall, thus favoring a closer approach of the incoming Ng interacting partner in the linear configuration and rationalizing the higher energy stability of the linear vs T-shaped isomer. A rough estimation of the polar flattening in the X_2_ series, therefore, is highly desirable, also enabling a fine calibration of the semi-empirical parameters in the analytical potential form. One possibility consists in the comparison of the electron density profile of the X_2_ molecules along two selected directions, namely the X_2_ bond axis and the plane perpendicular to it, at the fixed X nucleus position. The results are reported in Figure 6, focusing on the low electron density regions, in the outermost part of halogens, which are of interest of the polar flattening. For each dihalogen molecule, the electron density profile along the bond axis direction was systematically lower than that along the direction perpendicular to it, thus confirming the polar flatting of the electron density for each halogen atom in the X_2_ molecule. It is reasonable that the shift in the position of the repulsive wall in the linear isomers had to be strictly related to the shift at lower distance of the electron density profile along the X_2_ bond vs the plane perpendicular to it. A measure of the shift may be provided by the Δ*z* value, defined as the distance between two points of X_2_ density profiles at a selected density value. At the isodensity value of 1 me/bohr^3^, which is usually employed to map the Coulomb potential for the analysis of the σ-hole [20,21], the Δ*z* values were computed respectively as 0.23/0.25/0.25 Å for Cl_2_/Br_2_/I_2_, thus suggesting a comparable flattening of the electron density on the outer region of the covalent bond in the halogen series. The estimated polar flattening enabled a decrease at shorter distances of the repulsive wall position in the linear vs T-shaped isomer of about 4%–5%. 

### 2.5. Charge Displacement Analysis

**Figure 7:**
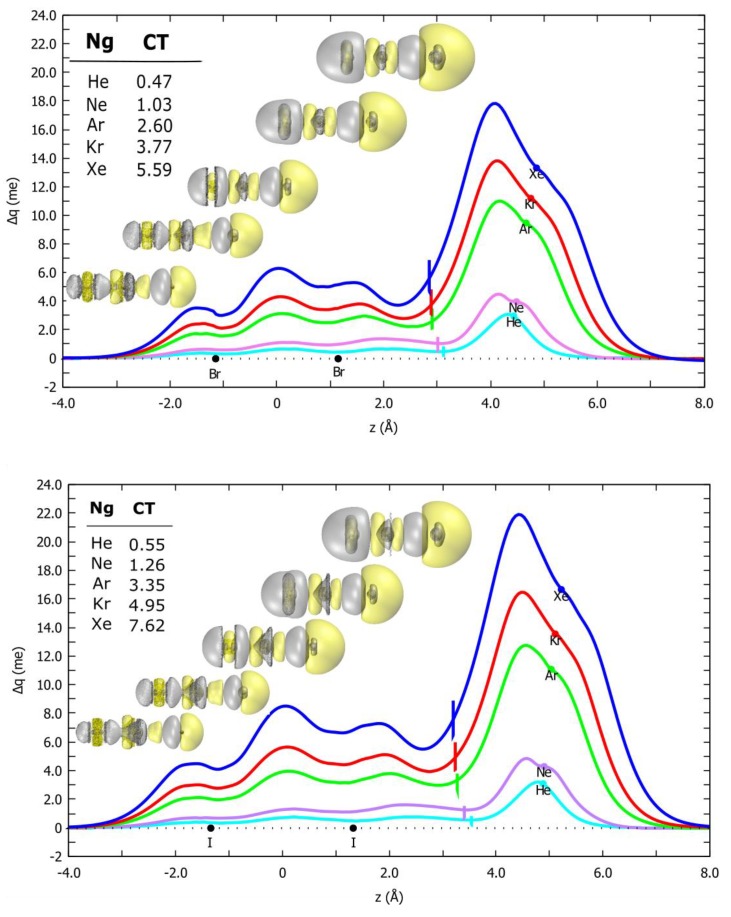
Charge–displacement (CD) curves of (*X* ^1^*Σ_g_*^+^) Ng–Br_2_ (top) and Ng–I_2_ (bottom) complexes in the linear configuration. Dots represent the atomic nuclei position on the z-axis and vertical lines mark the isodensity boundaries. Inset: 3D isodensity plots of the electron density change, accompanying bond formation (∆ρ = 8⋅× 10^−6^ me·Bohr^−3^, negative/positive values in yellow/silver); charge transfer (CT) values evaluated at the isodensity boundary.

The next subsection is addressed to characterization of the dependence of CT on the basic physical properties of the involved partners. This effort could be crucial to obtain a formulation of its stabilization contribution in terms of general validity. As previously done in the framework of our fine-tuned integrated experimental and theoretical approach [13,14,15], the presence of a CT component in the Ng–X_2_ bond, which is required by the PES model to quantitatively reproduce the experimental cross sections, was ascertained by resorting to the so-called CD function [22], which has proved especially useful for the identification and estimation of the CT in weakly bound systems [22,23,24,25]. The CD curves for selected Ng–X_2_ (X = Br, I) systems in the linear configuration are reported in Figure 7, together with the 3D contour plots of the electron density difference between the complex and the non-interacting fragments. The X_2_ substrate pronouncedly polarized the spherical cloud of the Ng center, which underwent a depletion/accumulation in the region opposite/towards X_2_. A visible amount of charge rearrangement was present even on the X_2_ moiety. Moreover, the CD curves were distinctly positive everywhere, indicating a corresponding electron charge flow in the direction from Ng to X_2_. This effect was a clear fingerprint of a donor role of Ng and was related to the presence of an XB interaction in the Ng–X_2_ complexes. Remarkably both the σ-hole and the electron density polar flattening on the halogen concurred to strengthen the CT in the linear isomer. For a plausible quantitative estimate of the amount of CT, we could take, as we have consistently done in previous works, the Δ*q* value at the so-called “isodensity boundary”, i.e., the point on the *z*-axis where the electron densities of the non-interacting fragments become equal. The results are detailed in the top left inset of Figure 7, and yielded comparable CT values for the Br_2_ and I_2_ complexes, ranging between 0.5 and 5.6–7.6 millielectron (me), going from the lighter to the heavier Ng. A similar trend was encountered for the Ng–Cl_2_ complexes (Ng = He, Ne, Ar) [13].

The CD analysis was also carried out on the selected Ar–Br_2_ complexes involving the Br_2_ molecule in the excited *B* ^3^*Π_u_* state, showing that the CT was essentially negligible, as already discussed for the Ng–Cl_2_ complexes [13].

### 2.6. CT Dependence on the First Ionization Potential

A proper rationalization of the increasing CT values along the Ng and X_2_ series in terms of the properties of the involved fragments is highly desirable, since the interaction CT component plays a key role in the determination of differences in the energy stability among the various conformers. We expect that the CT (and, consequently, the related energy amount, *V_CT_*) must be depending on the extent of the overlap between involved atomic/molecular orbitals. The associated contribution is ruled also by the energy separation between the states of the system coupled by CT. These effects can be modeled in terms of donor ionization energy and acceptor electron affinity, as they concur to determine both the dependence on the distance of the overlap integral, and of the separation energy between states of the system coupled by CT [26,27]. Taking into account that present systems, as many other weakly bounded adducts, are affected by non-resonant CT couplings, considered in the perturbation limit [24], a meaningful general relation, defining strength and range of the *V_CT_* component may be expressed as follows:

(1) $$V_{CT}\left(R\right)=\frac{B_{X}}{I_{Ng}-A_{X}-\frac{q^{2}}{R}}\cdot e^{-0.512\cdot \left(\sqrt{I_{Ng}}+\sqrt{A_{X}}\right)\cdot R}$$

For the present systems, *I_Ng_* is the donor first ionization energy and $A_{X}$ is the electron affinity of the electrophile X_2_. The numerator is correlated to the value of overlap integral between Ng and X_2_ orbitals, and, in particular, the pre-exponential term, $B_{X}$, returns information on the size of the X_2_ electron charge distribution. The denominator provides an estimate of the radial dependence of the energy difference between states coupled by CT, with the ($I_{Ng}-A_{X}$) term being the energy separation between the Ng–X_2_ and the Ng^+^–X_2_^−^ asymptotic energy, and the $\frac{q^{2}}{R}$ accounting for the Coulomb attraction between Ng^+^ and X_2_^−^ at the distance *R*.

It is quite instructive to compare the behavior of *V_CT_*, provided by Equation (1), with that gained from the semiempirical model (see Equation (9) in Section 3), for three series of Ng–X_2_ systems in the collinear configuration. In all cases *V_CT_* was evaluated at a selected intermediate distance value of R = 3 Å, here representing the separation distance between the Ng center and the X atom closer to Ng, being the only effectively involved in the CT from Ng to X_2_. The use of such condition is suitable to emphasize the *V_CT_* dependence on basic properties of the partners. Adopting Equation (1), the $B_{X}$ parameter was optimized in order to obtain the best comparison with the reference *V_CT_* contribution (also including the model uncertainties) gained from the semi-empirical parametrization (see below). 

In the comparison we also included the Ng–Cl adduct, which was previously investigated through state selected Cl atom beam experiments, carried out with the same molecular beam apparatus used here [26]. We report, in Table 3, the optimized parameters for the *V_CT_* formula (Equation (1)), together with the *I_Ng_* and $A_{X}$ values, as taken from the literature.

**Figure 8:**
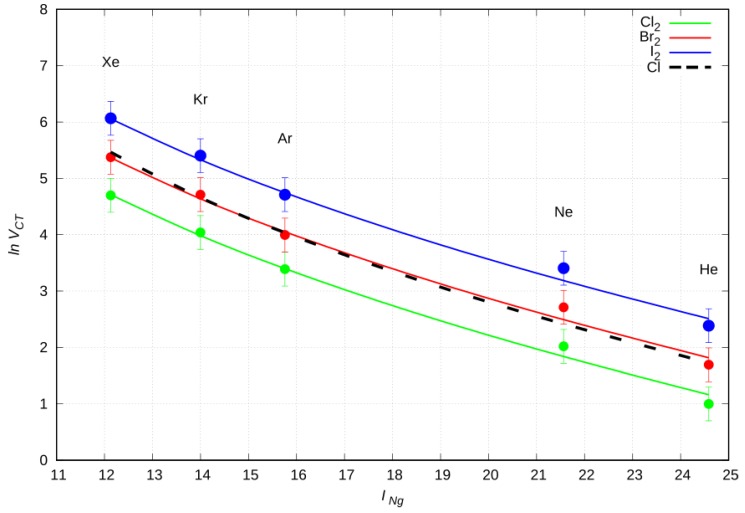
*Ln V_CT_* in the Ng–X_2_ complexes linear configurations vs Ng first ionization energy (*I_Ng_*, eV). Solid lines identify the values derived from Equation (1), while symbols with error bars refer to Equation (9); the dashed line refers to the Ng–Cl system.

From Figure 8, we notice that the semi-empirical function (Equation (1)) reproduces, within the error range, the calculated *V_CT_* values, suggesting the following trend: Ng–Cl_2_ < Ng–Cl ≃ Ng–Br_2_ < Ng–I_2_. This behavior can be explained introducing the *V_CT_* dependence on the dihalogens’ dimensions and electronic affinities: the more electrophilic they are, the greater their *V_CT_* component is. For the Cl atom, we have a different electronic deficit, because it has, properly, an electron missing on the *3p* orbital.

### 2.7. CT Proportional Constant

As a final important aspect of the CT component, contributing to the formation of the weak intermolecular XB, we briefly discuss the relation between the amount of electron charge transferred and the energy stabilization of the adduct arising from this interaction component. It is clear that, when CT is sufficiently small, as it was in the present cases, we should expect the energy stabilization *V_CT_* to be roughly proportional to CT, that is: *V_CT_* = *k* CT. Wang et al. [29] indeed found an excellent linear correlation. Obtaining a reliable estimate of the energy stabilization per transferred charge unit (*k*) is clearly a result of general interest, and the employed phenomenological–theoretical approach enables us to do just that.

**Figure 9:**
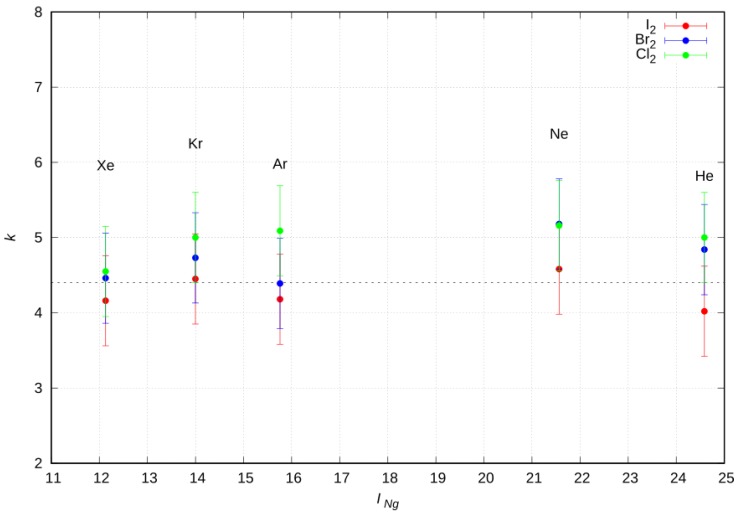
*k* values for each Ng–X_2_ system in the collinear isomer gained as ratio between the *V_CT_* strength, predicted by the semiempirical model at the ab initio optimized equilibrium distance, and the ab initio CT quantity, vs the Ngs first ionization energy (*I_Ng_*, eV).

We evaluated the *k* value for each Ng–X_2_ system in the collinear isomer as the ratio between the *V_CT_* strength, as predicted by the semiempirical model at the ab initio optimized equilibrium distance, and the ab initio CT quantity. The results, with their appropriate error bars, are shown in Figure 9 for the whole series of Ng elements, displayed on the scale of their ionization energy. The average proportional constant results were (4.6±1.0) eV/e, with the associated global error being estimated considering the combined uncertainty in the *V_CT_* (pre-exponential factor A_CT_ has a 15% error) and in the separation between the polar flattening and the CT stabilization contributions.

## 3. Materials and Methods

### 3.1. Phenomenological Approach

The phenomenological approach is solidly founded on the experiments carried out with the molecular beam technique, using the He, Ne, and Ar noble gases as projectiles and the electronic ground state Cl_2_/Br_2_ halogen molecules as targets. At given angle and energy resolution conditions, we measured the total integral cross-section *Q*(*v*) as a function of the collision velocity *v*, so that quantum “glory” interference effects were resolved. Unfortunately, on the present experimental apparatus, limit-angle problems prevented the measurement of the true quantum cross-section for heavier Kr or Xe beams. Similarly, substantial modifications of the apparatus and different operative conditions would be required to enable experiments with I_2_ targets.

Using the results from the measured integral cross-section *Q*(*v*) as a function of the collision velocity *v* (see Figure 1), integrated with other experimental data on various types of weakly bounded interacting systems, we proposed a well-defined and simple formulation of the intermolecular potential in the Ng–X_2_ systems. By denoting with *R* the distance of the Ng atom from the midpoint of the X_2_ bond (the latter assumed of fixed length) and with *Θ* the angle between **R** and the X_2_ bond axis (see Figure S1 in the SM), this formulation is as follows:

*V*(*R, Θ*) = *V_vdW_*(*R, Θ*) + *V*_3*B*_(*R, Θ*) + *V_CT_*(*R, Θ*) (2)

As previously ascertained, *V_vdW_* is best represented as the sum of individual interactions between Ng and each of the two X atoms (X*_a_* and X*_b_*), *V_vdW_* = *V_Ng–Xa_* + *V_Ng–Xb_*, each of them expressed as an improved Lennard–Jones (ILJ) function of the distance *r_i_* between Ng and X*_i_* and of the angle φ*_i_* between **r**_i_ and the X_2_ axis (see Figure S1 in SM):

(3) $$V_{Ng-X_{i}}\left(r_{i},\varphi_{i}\right)=\varepsilon \left(\varphi_{i}\right)\left[\frac{6}{n\left(r_{i},\varphi_{i}\right)}\cdot {\left(\frac{r_{m}\left(\varphi_{i}\right)}{r_{i}}\right)}^{n\left(r_{i},\varphi_{i}\right)}-\;\frac{n\left(r_{i},\varphi_{i}\right)}{n\left(r_{i},\varphi_{i}\right)-6}\cdot {\left(\frac{r_{m}\left(\varphi_{i}\right)}{r_{i}}\right)}^{6}\right].$$

Here, the $\varepsilon \left(\varphi_{i}\right)$ and $r_{m}\left(\varphi_{i}\right)$ parameters (potential energy minimum and equilibrium distance, respectively) are calculated as follows:

(4) $$\varepsilon \left(\varphi_{i}\right)=\;\varepsilon_{\left|\right|}\cdot cos^{2}\left(\varphi_{i}\right)+\varepsilon_{\cdot }\cdot sin^{2}\left(\varphi_{i}\right)$$

(5) $$r_{m}\left(\varphi_{i}\right)=\;r_{m||}\cdot cos^{2}\left(\varphi_{i}\right)+r_{m\cdot }\cdot sin^{2}\left(\varphi_{i}\right)$$

where the || and ⊥ symbols refer to the collinear ($\varphi_{i}=0$) and perpendicular ($\varphi_{i}=\pi /2$) configurations, respectively. The factor $n\left(r_{i},\varphi_{i}\right)$, which modulates the fall-off of the repulsion and the radial dependence of the intermediate and long-range attraction, depends on *β*, an additional parameter related to the “hardness” of the interacting partners, as follows:

(6) $$n\left(r_{i},\varphi_{i}\right)=\beta +4\cdot {\left(\frac{r_{i}}{r_{m}\left(\varphi_{i}\right)}\right)}^{2}$$

Here, *β* is optimally found to lie in the interval 7.0–7.5 for all atom pairs. The partial long-range dispersion attraction coefficients are described only by the asymptotic *V_vdW_* component of the potential and, according to the ILJ formulation, their expression is:

(7) $$C_{6i}=\left[\varepsilon \left(\varphi_{i}\right)\cdot r_{m}^{6}\left(\varphi_{i}\right)\right]$$

with the global attraction coefficient resulting as the sum of the two angularly averaged *C_6i_* terms.

The values of the ε and the $r_{m}$ parameters were predicted in an internally consistent way for all Ng–X_2_ systems from the polarizability components (see refs 10–14 and references therein), and they were validated by comparing the calculated cross-sections with the experimental results. During the analysis, an additional constraint of providing global average asymptotic attractions was imposed in order to obtain a satisfactory agreement (within about 10%) with the results reported in reference [30]. 

As previously done for other systems involving halogen atoms [13,31,32], the zero order values of $r_{m||}$ were decreased by about 4% to account for the polar flattening effect in the ground state of X_2_. Such effect is related to the peculiarity of the electronic charge distribution of the X atom in the direction pointing at the approaching Ng atom in the collinear isomer, the origin and details of which are rationalized in the theoretical sections of this paper. The reduction of $r_{m||}$ must be accompanied by corresponding increase of $\varepsilon_{\left|\right|}$ in order to maintain the $C_{6}$ coefficient constant.

The second term, *V*_3*B*_, in the expression for V(R, Θ), can be conveniently formulated as:

(8) $$V_{3B}\left(R,\;\Theta \right)=A_{3B}\cdot sin^{2}\left(2\Theta \right)e^{-3.0\cdot R}$$

This term is crucial to properly represent the angular dependence of the full PES, especially in the proximity of the saddle point between the collinear and perpendicular configurations, where the molecular repulsion by occupied π* orbitals is more prominent. With respect to a previous formulation [11], the expression for this term was slightly improved to guarantee a smooth second derivative in vicinity of *Θ* = π/2.

Finally, the charge-transfer term of the potential formulation was defined as:

(9) $$V_{CT}\left(R,\Theta \right)=-\underset{i=a,b}{\sum }A_{CT}\cdot cos^{4}\left(\varphi_{i}\right)\cdot e^{-3.0\cdot r_{i}}$$

The dynamical treatment used for the scattering data analysis was extensively described in recent papers. It affords a satisfactory reproduction of the measured *Q*(*v*) (full lines in Figure 1) by essentially only varying the parameters *A*_3*B*_ and *A_CT_*, while keeping the other parameters fixed at their predicted value to within very small adjustments.

The modulation of the involved potential parameters was gained through empirical/semi-empirical formulas developed in our laboratory [10,11,12,13,14,27,28], which represent the strength of the basic intermolecular interaction components in terms of fundamental physical properties of the involved partners, such as the electronic polarizabilities, which define strength, range, and anisotropy of the *V_vdW_* component. Such formulas also account for the dependence of specific parameters on the ionization potential of Ng and on the π*⟶σ* electronic excitation of X_2_, which is accompanied by a substantial electronic charge rearrangement, causing an increase of the molecular polarizability and of its anisotropy.

By then comparing the model predictions with the results of ab initio calculations, reported in the previous section, the potential parameters can be further refined and thereby the overall potential formulation exhaustively validated for the entire family of Ng–X_2_ adducts, with X_2_ either in its ground or first excited electronic state. The parameters values derived for the analytical form of the Ng–X_2_ PESs are reported in Table 4 and Table 5.

### 3.2. Computational Details

The ab initio calculations were performed with the MOLPRO program [33]. Since the investigated non-covalent interactions have a strong dispersion component, it is fundamental that the computational method used to treat a HX system is of highly accurate level, adequately accounting for the consistent electron correlation. Accordingly, the calculations were carried out at the coupled-cluster level of theory [34], with single, double, and perturbatively included triple excitations, CCSD(T), using augmented correlation consistent polarized valence triple- (only in the He case), quadruple-, quintuple-ζ basis sets (aug-cc-PVXZ, hereafter labelled as AVXZ, with X = T, Q, 5). Selected cuts of the ground state PESs for the Ng–X_2_ (X = Br, I) complexes were investigated by considering the Ng atom in the ^1^*S*_0_ ground state and the Cl_2_ molecule in the (*X*^1^ *Σ_g_*^+^) ground state. We also considered the Ar–Br_2_ complex with Br_2_ being in its first excited state (*B^3^Π_u_*), characterized by a (π_g_*)^3^(σ_u_*)^1^ valence shell configuration. Remarkably, the double degeneracy of the *Π* state was lifted upon approach of the Ng atom. The overall symmetry was lowered to the C_2v_ (C_s_) point groups for the T-shaped (saddle) isomer, and led to two electronic states with A_1_/B_2_ (A’/A’’) symmetries, which became degenerate for the collinear geometry.

According to the formulation of the phenomenological PES, we used the Jacobi coordinates (*R*, *r*, *Θ*) to describe the triatomic Ng–X_2_ complexes. The Br–Br (I–I) bond distance in the adducts was fixed at the equilibrium value 2.28 Å (2.666 Å) in the isolated Br_2_ (I_2_) molecule in the ground state [35], since the relatively weak interaction with Ng was expected to leave the geometry of X_2_ essentially unaffected. For the Br_2_ molecule, the first excited state, an equilibrium distance of 2.699 Å was considered [36]. For each Ng–X_2_ complex, we varied the intermolecular distance, *R*, in the range 2.0–7.0 Å, for both *Θ* = 0° and 90° values. For the *X* ground state, we also examined peculiar cuts of the Ng–X_2_ PESs by varying the *Θ* angle in the range 0–90° (step by 5°) and optimizing the intermolecular distance *R*. In this case, the *Θ* value corresponding to the energy maximum along the path returned the saddle configuration between the two limiting ones. 

The basis set superposition errors (BSSEs) on the interaction energy values computed with the AVXZ basis sets were evaluated by applying the counterpoise method [37,38].

### 3.3. Charge Displacement Function

As the above discussion implies, specific configurations of Ng–X_2_ (X=Br,I) may be selectively stabilized when there is a CT component. A well-established method that permits in a simple yet powerful way to access and quantify the electron displacement occurring upon formation of the bond is the CD function [22]. This function gives, at each point *z* along an axis joining two interacting fragments, the electron charge (Δq) that, upon formation of the complex, is displaced from right to left across the plane perpendicular to the axis through *z*. Its expression is:

(10) $$\Delta q\left(z\right)=\;\int_{-\infty }^{+\infty }dx\;\int_{-\infty }^{+\infty }dy\;\int_{-\infty }^{z}\Delta \rho \left(x,y,z^{\prime }\right)dz^{\prime }$$

where the integrand Δρ is the electron density difference between the complex under study and its non-interacting fragments, i.e., the change in electron density brought about by the interaction.

The CD curve provides in most cases a straightforward and unambiguous tool to assess the presence and extent of CT in the formation of the adduct, especially in few-atom systems such as the ones at hand. If the function is appreciably different from zero and does not change in sign in the region between the fragments, we can with confidence assert that CT is taking place. Conversely, if the curve crosses zero in this region, CT may be uncertain (both in magnitude and direction). When CT is ascertained, it is useful, for comparative purposes, to obtain a definite numerical estimate of it, by considering the CD function value at a specific point between the fragments along the z axis. In this case, as in previous ones [13,14,15], we chose as fragment separator the point along *z* at which the electron densities of the non-interacting fragments become equal (isodensity boundary). 

## 4. Conclusions

In the present manuscript we have characterized by a thoroughly integrated experimental and theoretical approach the XB interaction in the series of prototype noble gas–dihalogen adducts. We gain an internally consistent description for the interaction in all the Ng–X_2_ families (Ng = He, Ne, Ar, Kr, Xe; X_2_ = Cl_2_, Br_2_, I_2_), together with an analytical formulation of all the PESs, which is fine tuned by a few well-defined physical parameters. The nature of the interaction is strongly anisotropic not only in the X_2_ ground state, but also for the excited one. In the Ng–X_2_ ground state an interaction with XB characteristic clearly emerges limited to the linear configuration, as suggested by the polar flattening of the X_2_ electron density along the X–X bond direction and by the presence of a sizable CT accompanying the adduct formation. These peculiar features are missing in the T-shape orientation adducts or in the linear complexes involving the X_2_ excited state, so that the interaction has substantially a vdW nature. The analytical potential formulation based on the investigated prototype Ng–X_2_ adducts can be easily extended to more complex systems, such as those involving the H_2_O or NH_3_ polyatomic molecules as interacting partners. Remarkably, in the latter cases a more pronounced anisotropy character is expected in the XB interaction, being affected by the interacting partner orientation.

## Figures and Tables

**Table 1:** Equilibrium distances (*R_m_*, Å) and binding energy values (*E_m_*, meV; 1 meV = 0.02306 kcal/mol) of the phenomenological interaction potential considered as spherical average (*V_sph_*) or in the selected linear (*V_lin_*) and perpendicular (*V_perp_*) orientation for the noble gas–dihalogen molecule (Ng–X_2_) complexes in the *X* ^1^*Σ_g_* ground state.

	*V_sph_*		*V_lin_*		*V_perp_*	
	*R_m_*	*E_m_*	*R_m_*	*E_m_*	*R_m_*	*E_m_*
**He–Cl_2_**	4.16	3.23	4.27	5.93	3.39	6.15
**Ne–Cl_2_**	4.16	6.98	4.29	12.21	3.44	12.10
**Ar–Cl_2_**	4.33	17.99	4.53	27.01	3.70	27.12
**Kr–Cl_2_**	4.43	23.01	4.61	34.67	3.83	32.78
**Xe–Cl_2_**	4.56	27.77	4.73	41.45	4.00	37.71
**He–Br_2_**	4.41	3.24	4.51	6.51	3.50	6.43
**Ne–Br_2_**	4.40	7.29	4.52	14.09	3.54	13.22
**Ar–Br_2_**	4.57	18.42	4.75	30.03	3.81	29.28
**Kr–Br_2_**	4.61	25.69	4.77	43.92	3.88	38.91
**Xe–Br_2_**	4.73	32.24	4.86	55.02	4.04	46.18
**He–I_2_**	4.85	2.76	4.95	5.54	3.79	5.54
**Ne–I_2_**	4.80	6.46	4.90	13.21	3.78	12.21
**Ar–I_2_**	4.88	19.20	5.04	34.66	3.94	32.98
**Kr–I_2_**	4.93	26.34	5.05	49.82	4.02	43.19
**Xe–I_2_**	5.02	34.64	5.11	65.77	4.16	54.26

**Table 2:** Equilibrium distances (*R_m_*, Å) and binding energy values (*E_m_*, meV; 1 meV = 0.02306 kcal/mol) of the phenomenological interaction potential considered as spherical average (*V_sph_*) or in the selected linear (*V_lin_*) and perpendicular (*V_perp_*) orientation for the Ng–X_2_ complexes in the *B* ^3^*Π_u_* excited state.

	*V_sph_*		*V_lin_*		*V_perp_*	
	*R_m_*	*E_m_*	*R_m_*	*E_m_*	*R_m_*	*E_m_*
**He–Cl_2_**	4.37	2.69	4.82	3.24	3.37	5.79
**Ne–Cl_2_**	4.40	5.52	4.84	6.77	3.41	11.59
**Ar–Cl_2_**	4.58	14.45	5.00	17.60	3.67	27.37
**Kr–Cl_2_**	4.68	18.66	5.10	22.68	3.78	34.18
**Xe–Cl_2_**	4.82	23.18	5.25	28.11	3.95	40.61
**He–Br_2_**	4.57	2.67	5.03	3.15	3.61	5.37
**Ne–Br_2_**	4.58	5.66	5.03	6.77	3.63	11.21
**Ar–Br_2_**	4.73	16.01	5.16	19.24	3.82	29.41
**Kr–Br_2_**	4.81	21.25	5.24	25.43	3.92	38.03
**Xe–Br_2_**	4.93	27.14	5.35	32.51	4.07	46.35
**He–I_2_**	5.12	2.13	5.62	2.85	3.83	4.88
**Ne–I_2_**	5.10	4.66	5.59	6.32	3.82	10.62
**Ar–I_2_**	5.19	14.69	5.66	20.05	3.94	31.73
**Kr–I_2_**	5.26	20.15	5.73	27.30	4.02	42.49
**Xe–I_2_**	5.35	26.94	5.83	36.42	4.14	55.01

**Table 3:** Ng first ionization potential energies (*I_Ng_*), X/X_2_ electron affinities $\left(A_{X}\right)$ and $B_{X}$ optimized parameters.

	*I_Ng_* (eV) ^(a)^		$A_{X}\;(\mathrm{eV})\;{}^{\left(a\right)}$	$B_{X}$ $[\left(\mathrm{eV}{)}^{2}\right]\;{}^{\left(b\right)}$
**He**	24.587	**Cl**	3.621	3430
**Ne**	21.564	**Cl_2_**	2.38 ± 0.1	1300
**Ar**	15.759	**Br_2_**	2.55 ± 0.1	2500
**Kr**	13.999	**I_2_**	2.55 ± 0.05	5000
**Xe**	12.130			

^(a)^ Values from reference [28]; ^(b)^ Absolute error of ±10%.

**Table 4:** Potential parameters (*r_m_* in Å; *ε*, *Α_C__Τ_*, *A*_3*B*_ in meV) employed for the formulation of the Ng–Cl atom–atom pairwise interaction for Ng–X_2_ systems in the (*X* ^1^*Σ_g_*^+^) ground state. || and ⊥ symbols refer to the collinear and perpendicular configurations, respectively. β is equal to 7.0 except for in Ng–Cl_2_ systems (β = 7.5). The maximum estimated uncertainty is about 10% for *ε*, 3% for *r_m_*, and 10%–15% for *A_CT_* and *A*_3*B*_.

	*r_m_*||	*ε* ||	*r_m_*⊥	*ε* ⊥	*A_CT_*	*A*_3*B*_
**He–Cl_2_**	3.39	4.47	3.55	2.93	310,000	22,000
**Ne–Cl_2_**	3.44	8.76	3.60	5.76	450,000	61,000
**Ar–Cl_2_**	3.68	19.70	3.85	13.00	700,000	240,000
**Kr–Cl_2_**	3.79	24.40	3.98	15.70	830,000	460,000
**Xe–Cl_2_**	3.94	28.20	4.14	18.10	970,000	890,000
**He–Br_2_**	3.53	4.71	3.71	3.02	620,000	44,000
**Ne–Br_2_**	3.57	9.66	3.75	6.21	900,000	120,000
**Ar–Br_2_**	3.80	21.30	4.00	13.90	1,400,000	440,000
**Kr–Br_2_**	3.87	28.90	4.07	18.40	1,660,000	900,000
**Xe–Br_2_**	4.01	34.50	4.23	21.90	1,900,000	1,750,000
**He–I_2_**	3.81	4.00	4.06	2.59	1,550,000	88,000
**Ne–I_2_**	3.81	8.76	4.05	5.70	2,250,000	244,000
**Ar–I_2_**	3.95	23.60	4.20	15.50	3,500,000	900,000
**Kr–I_2_**	4.03	30.90	4.28	20.30	4,150,000	1,800,000
**Xe–I_2_**	4.16	38.60	4.41	25.60	4,750,000	3,500,000

**Table 5:** Potential parameters (*r_m_* in Å; *ε*, *Α_C__Τ_, A*_3*B*_ in meV) employed for the formulation of the Ng–Cl atom–atom pairwise interaction for Ng–X_2_ systems in the (*B^3^Π_u_^+^*) excited state. || and ⊥ symbols refer to the collinear and perpendicular configurations, respectively. β is equal to 7.0 except for in Ng–Cl_2_ systems (β = 7.5). The maximum estimated uncertainty is about 10% for *ε* and 3% for *r_m_*.

	*r_m_*||	*ε*||	*r_m_*⊥	*ε*⊥	*A_CT_*	*A*_3*B*_
**He–Cl_2_**	3.62	3.03	3.58	2.88	0	0
**Ne–Cl_2_**	3.64	6.32	3.62	5.73	0	0
**Ar–Cl_2_**	3.81	16.30	3.87	13.40	0	0
**Kr–Cl_2_**	3.91	20.90	3.98	16.70	0	0
**Xe–Cl_2_**	4.06	25.70	4.14	19.80	0	0
**He–Br_2_**	3.83	2.92	3.81	2.66	0	0
**Ne–Br_2_**	3.83	6.27	3.83	5.53	0	0
**Ar–Br_2_**	3.96	17.70	4.02	14.40	0	0
**Kr–Br_2_**	4.04	23.30	4.11	18.60	0	0
**Xe–Br_2_**	4.16	29.60	4.26	22.60	0	0
**He–I_2_**	4.12	2.70	4.12	2.40	0	0
**Ne–I_2_**	4.09	6.00	4.11	5.20	0	0
**Ar–I_2_**	4.16	19.00	4.23	15.40	0	0
**Kr–I_2_**	4.23	25.80	4.31	20.60	0	0
**Xe–I_2_**	4.33	34.30	4.42	26.60	0	0

## Supplementary Materials

The following are available online, Figure S1: Coordinate systems for the Ng-X_2_ systems case study; Figure S2: Angular MEP for the Ng-Cl_2_ complexes in the ground *X* state of Cl_2_, reporting the interaction energy, as derived from the potential parametrization, vs the angular variable Θ; Figure S3: Angular MEP for the Ng-Cl_2_ complexes in the excited *B* state of Cl_2_, reporting the interaction energy, as derived from the potential parametrization, vs the angular variable Θ; Figure S4: Angular MEP for the Ng-I_2_ complexes in the ground *X* state of I_2_, reporting the interaction energy, as derived from the potential parametrization, vs the angular variable Θ; Figure S5: Angular MEP for the Ng-I_2_ complexes in the excited *B* state of I_2_, reporting the interaction energy, as derived from the potential parametrization, vs the angular variable Θ; Figure S6: CCSD(T) potential energy curves (interaction potential *V* vs. Ng-Br_2_ distance R) for the ground state Ng-Br_2_ (AV5Z) and Ng-I_2_ (AVQZ) complexes; Figure S7: Charge displacement functions (CCSD/AVQZ) of Ar-X_2_ (*X* ^1^*Σ_g_*^+^) complexes in the linear configuration. Dots on graphs represent the position in the z-axis of the different atoms nuclei. Vertical lines are the isodensity boundary; Table S1: Equilibrium distances R_m_ (Å) and binding energy values E_m_ (meV) for Ng-Br_2_ complexes in both ground (*X* ^1^*Σ_g_*^+^) [CCSD(T)/AVXZ, X = T, Q, 5] and excited (*B* ^3^*Π_u_*) [UCCSD(T)/AVTZ] states in the three relative configurations (linear, T-shaped, saddle). In parenthesis the energy values with BSSE corrections; Table S2: V_CT_ components and CT constant k evaluated at the optimized equilibrium distances ($R_{Ng-X_{2}}$, Å).

## References

[1] Desiraju G.R., Ho P.S., Kloo L., Legon A.C., Marquardt R., Metrangolo P., Politzer P., Resnati G., Rissanen K. (2013). Definition of the halogen bond (iupac recommendations 2013). Pure Appl. Chem..

[2] Han Z.M., Czap G., Chiang C.L., Xu C., Wagner P.J., Wei X.Y., Zhang Y.X., Wu R.Q., Ho W. (2017). Imaging the halogen bond in self-assembled halogenbenzenes on silver. Science.

[3] Gilday L.C., Robinson S.W., Barendt T.A., Langton M.J., Mullaney B.R., Beer P.D. (2015). Halogen Bonding in Supramolecular Chemistry. Chem. Rev..

[4] Rohrbacher A., Halberstadt N., Janda K.C. (2000). The Dynamics of Noble Gas-Halogen Molecules and Clusters. Annu. Rev. Phys. Chem..

[5] Beswick J.A., Halberstadt N., Janda K.C. (2012). Structure and Dynamics of Noble Gas-Halogen and Noble Gas Ionic Clusters: When Theory Meets Experiment. Chem. Phys..

[6] Wei J., Makarem C., Reinitz A.L., Darr J.P., Loomis R.A. (2012). Accurate Measurement of the T-Shaped And Linear Ar··· I_2_(X, ν″=0) Binding Energies Using Vibronic-Specific I_2_(B, ν) Fragment Velocity-Map Imaging. Chem. Phys..

[7] Delgado-Tellez L., Valdes A., Prosmiti R., Villarreal P., Delgado-Barrio G. (2011). Ab Initio Characterization of the Ne-I_2_ Van Der Waals Complex: Intermolecular Potentials and Vibrational Bound States. J. Chem. Phys..

[8] Baturo V.V., Lukashov S.S., Poretsky S.A., Pravilov A.M. (2017). The RgI_2_ (Ion-Pair States) van der Waals Complexes. Eur. Phys. J. D.

[9] Legon A.C. (2010). The Halogen Bond: An Interim Perspective. Phys. Chem. Chem. Phys..

[10] Politzer P., Murray J.S., Clark T. (2015). Mathematical modeling and physical reality in noncovalent interactions. J. Mol. Modeling.

[11] Shields Z.P., Murray J.S., Politzer P. (2010). Directional Tendencies of Halogen and Hydrogen Bonds. Int. J. Quantum Chem..

[12] Pirani F., Cappelletti D., Falcinelli S., Cesario D., Nunzi F., Belpassi L., Tarantelli F. (2019). Selective Emergence of Halogen Bond in Ground and Excited States of Noble-Gas—Chlorine Systems. Angew. Chem. Int. Ed..

[13] Nunzi F., Cesario D., Belpassi L., Tarantelli F., Roncaratti L.F., Falcinelli S., Cappelletti D., Pirani F. (2019). Insight into The Halogen-Bond Nature Of Noble Gas-Chlorine Systems By Molecular Beam Scattering Experiments, Ab Initio Calculations And Charge Displacement Analysis. Phys. Chem. Chem. Phys..

[14] De Santis M., Nunzi F., Cesario D., Belpassi L., Tarantelli F., Cappelletti D., Pirani F. (2018). Cooperative Role of Halogen And Hydrogen Bonding In The Stabilization Of Water Adducts With Apolar Molecules. New J. Chem..

[15] Cappelletti D., Aquilanti V., Bartocci A., Nunzi F., Tarantelli F., Belpassi L., Pirani F. (2016). Interaction of O_2_ with CH_4_, CF_4_, and CCl_4_ by Molecular Beam Scattering Experiments and Theoretical Calculations. J. Phys. Chem. A.

[16] Cappelletti D., Ronca E., Belpassi L., Tarantelli F., Pirani F. (2012). Revealing Charge-Transfer Effects in Gas-Phase Water Chemistry. Acc. Chem. Res..

[17] Cappelletti D., Cinti A., Nicoziani A., Falcinelli S., Pirani F. (2019). Molecular Beam Scattering Experiments as a Sensitive Probe of the Interaction in Bromine-Noble Gas Complexes. Front. Chem..

[18] Pirani F., Vecchiocattivi F. (1982). A Fast and Accurate Semiclassical Calculation of the Total Elastic Cross Section In The Glory Energy Range. Mol. Phys..

[19] Pirani F., Brizi S., Roncaratti L.F., Casavecchia P., Cappelletti D., Vecchiocattivi F. (2008). Beyond the Lennard-Jones Model: A Simple and Accurate Potential Function Probed by High Resolution Scattering Data Useful For Molecular Dynamics Simulations. Phys. Chem. Chem. Phys..

[20] Clark T., Hennemann M., Murray J.S., Politzer P. (2007). Halogen Bonding: The σ-hole. J. Mol. Modeling.

[21] Riley K.E., Murray J.S., Fanfrlik J., Rezac J., Sola R.J., Concha M.C., Ramos F.M., Politzer P. (2013). Halogen bond tunability II: The varying roles of electrostatic and dispersion contributions to attraction in halogen bonds. J. Mol. Modeling.

[22] Belpassi L., Infante I., Tarantelli F., Visscher L. (2008). The Chemical Bond Between Au(I) and the Noble Gases. Comparative Study of NgAuF And NgAu^+^ (Ng = Ar, Kr, Xe) By Density Functional and Coupled Cluster Methods. J. Am. Chem. Soc..

[23] Bistoni G., Belpassi L., Tarantelli F., Pirani F., Cappelletti D. (2011). Charge-Displacement Analysis of the Interaction in the Ammonia-Noble Gas Complexes. J. Phys Chem.

[24] Nunzi F., Cesario D., Pirani F., Belpassi L., Frenking G., Grandinetti F., Tarantelli F. (2017). Helium Accepts Back-Donation In Highly Polar Complexes: New Insights into the Weak Chemical Bond. J. Phys. Chem. Lett..

[25] Nunzi F., Cesario D., Pirani F., Belpassi L., Tarantelli F. (2018). Modelling Charge Transfer in Weak Chemical Bonds: Insights from the Chemistry of Helium. Chemphyschem.

[26] Aquilanti V., Cappelletti D., Lorent V., Luzzatti E., Pirani F. (1992). The Ground and Lowest Excited-States of XeCl By Atomic-Beam Scattering. Chem. Phys. Lett..

[27] Pirani F., Giulivi A., Cappelletti D., Aquilanti V. (2000). Coupling by Charge Transfer: Role In Bond Stabilization For Open-Shell Systems And Ionic Molecules And In Harpooning And Proton Attachment Processes. Mol. Phys..

[28] Weast R.C., Astle M.J., Beyer W.H. (1988). CRC Handbook of Chemistry and Physics.

[29] Wang C., Danovich D., Mo Y., Shaik S. (2014). On the Nature of the Halogen Bond. J. Chem. Theory Comput..

[30] Olney T.N., Cann N.M., Cooper G., Brion C.E. (1997). Absolute scale determination for photoabsorption spectra and the calculation of molecular properties using dipole sum rules. Chem. Phys..

[31] Cappelletti D., Bartocci A., Grandinetti F., Falcinelli S., Belpassi L., Tarantelli F., Pirani F. (2015). Experimental Evidence of Chemical Components in the Bonding of Helium and Neon with Neutral Molecules. Chem. A Eur. J..

[32] Bartocci A., Belpassi L., Cappelletti D., Falcinelli S., Grandinetti F., Tarantelli F., Pirani F. (2015). Catching the Role of Anisotropic Electronic Distribution and Charge Transfer in Halogen Bonded Complexes of Noble Gases. J. Chem. Phys..

[33] Werner H.J., Knowles P.J., Knizia G., Manby F.R., Schutz M. (2012). Molpro: A General-Purpose Quantum Chemistry Program Package. Wiley Interdiscip. Rev. Comput. Mol. Sci..

[34] Raghavachari K., Trucks G.W., Pople J.A., Headgordon M. (1989). A 5th-Order Perturbation Comparison of Electron Correlation Theories. Chem. Phys. Lett..

[35] Huber K.P., Herzberg G. (1979). Molecular Spectra and Molecular Structure, Constants of Diatomic Molecules, Vol. IV.

[36] Le Roy R.J., Bernstein R.B. (1971). Dissociation Energies and Long-Range Potentials of Diatomic Molecules from Vibrational Spacings: The Halogens. J. Mol. Spectrosc..

[37] Jansen H.B., Ros P. (1991). Non-Empirical Molecular Orbital Calculations on the Protonation of Carbon Monoxide. Chem. Phys. Lett..

[38] Liu B., McLean A.D. (1973). Accurate Calculation of the Attractive Interaction of Two Ground State Helium Atoms. J. Chem. Phys..

