# Supplementary material for: The Halogen-Bond Nature in Noble Gas–Dihalogen Complexes from Scattering Experiments and Ab Initio Calculations
Source: Molecules. 2019 Nov 23;24(23):4274. doi: 10.3390/molecules24234274 (PMC6930525; doi:10.3390/molecules24234274)
Supplement: Supplementary file 1 [file molecules-24-04274-s001.pdf]

The Halogen-Bond Nature in Noble Gas-Dihalogen Complexes from Scattering Experiments and Ab-Initio Calculations

Francesca Nunzi [1,2,*], Benedetta Di Erasmo [1], Francesco Tarantelli [1,2], David Cappelletti [1] and Fernando Pirani [1,*]

[1] Dipartimento di Chimica, Biologia e Biotecnologie, via Elce di Sotto 8, I-06123 Perugia, Italy; benedetta.dierasmo@studenti.unipg.it (B.D.E.); francesco.tarantelli@unipg.it (F.T.); david.cappelletti@unipg.it (D.C.)

[2] Istituto CNR di Scienze e Tecnologie Chimiche "Giulio Natta" (CNR-SCITEC), via Elce di Sotto, I-06123 Perugia, Italy

* Correspondence: francesca.nunzi@unipg.it (F.N.); fernando.pirani@unipg.it (F.P.)

Supporting Materials

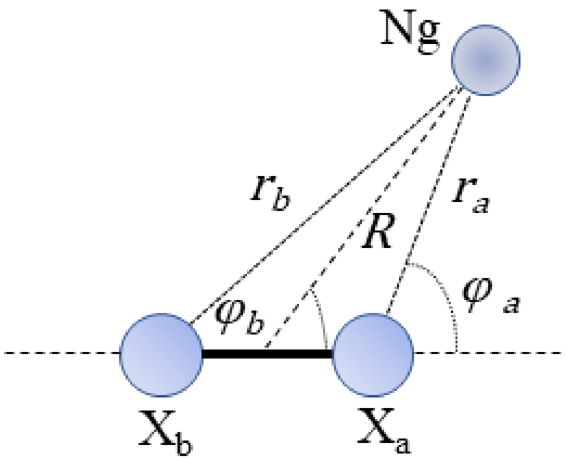

Figure S1. Coordinate systems for the Ng-X_2 systems case study.

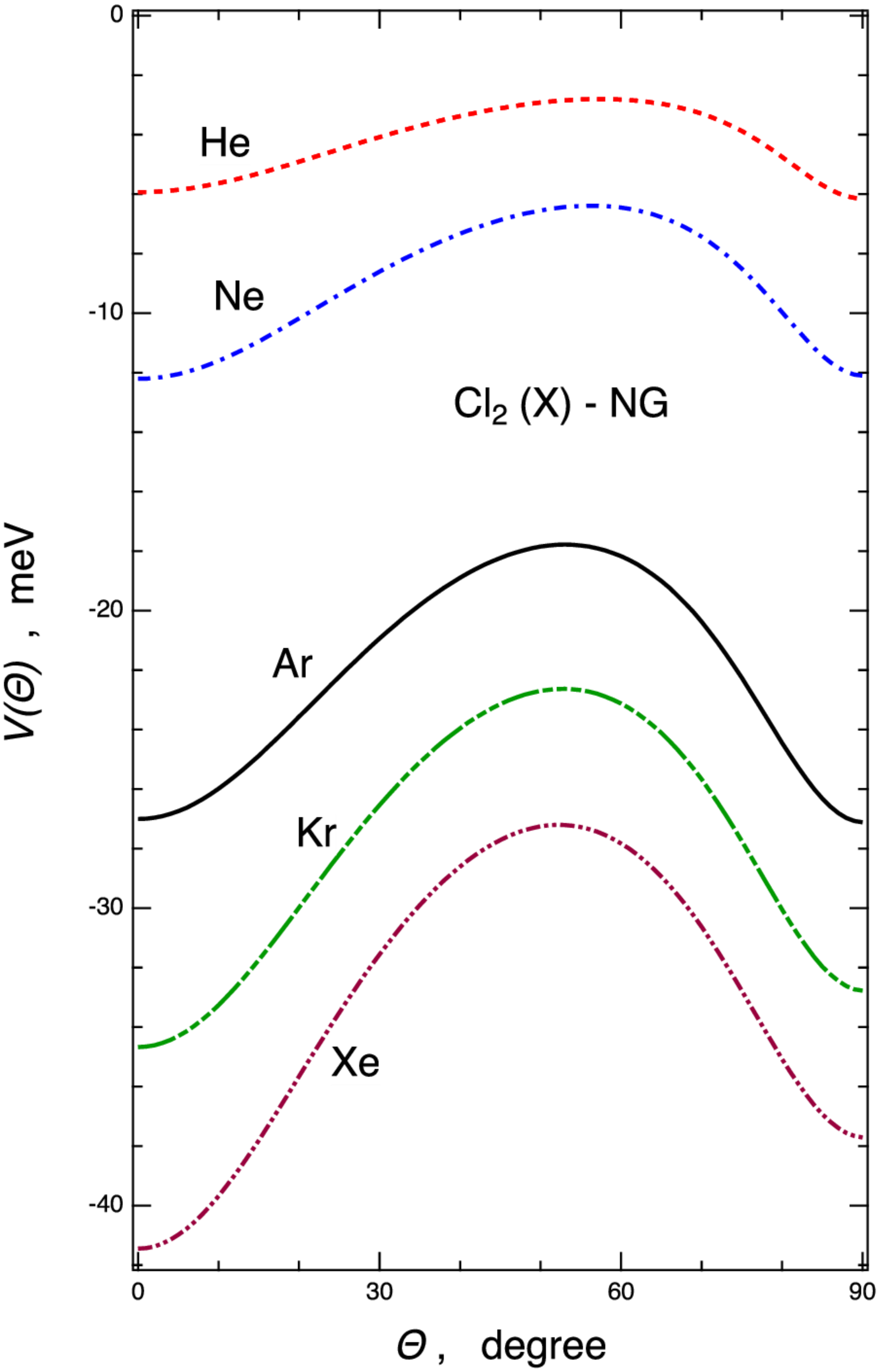

Figure S2. Angular MEP for the Ng-Cl_2 complexes in the ground *X* state of Cl_2, reporting the interaction energy, as derived from the potential parametrization, vs the angular variable Θ.

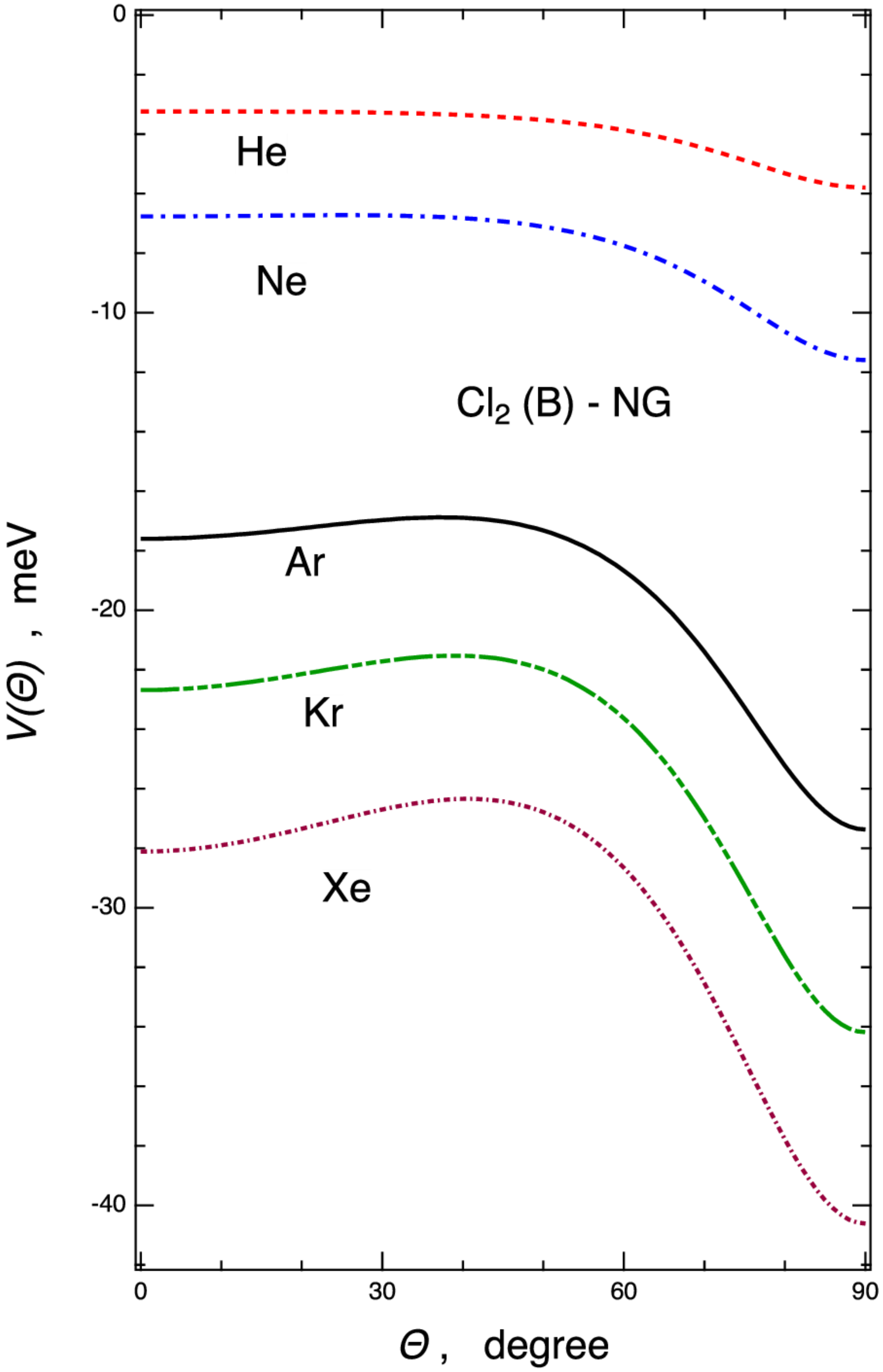

Figure S3. Angular MEP for the Ng-Cl_2 complexes in the excited *B* state of Cl_2, reporting the interaction energy, as derived from the potential parametrization, vs the angular variable Θ.

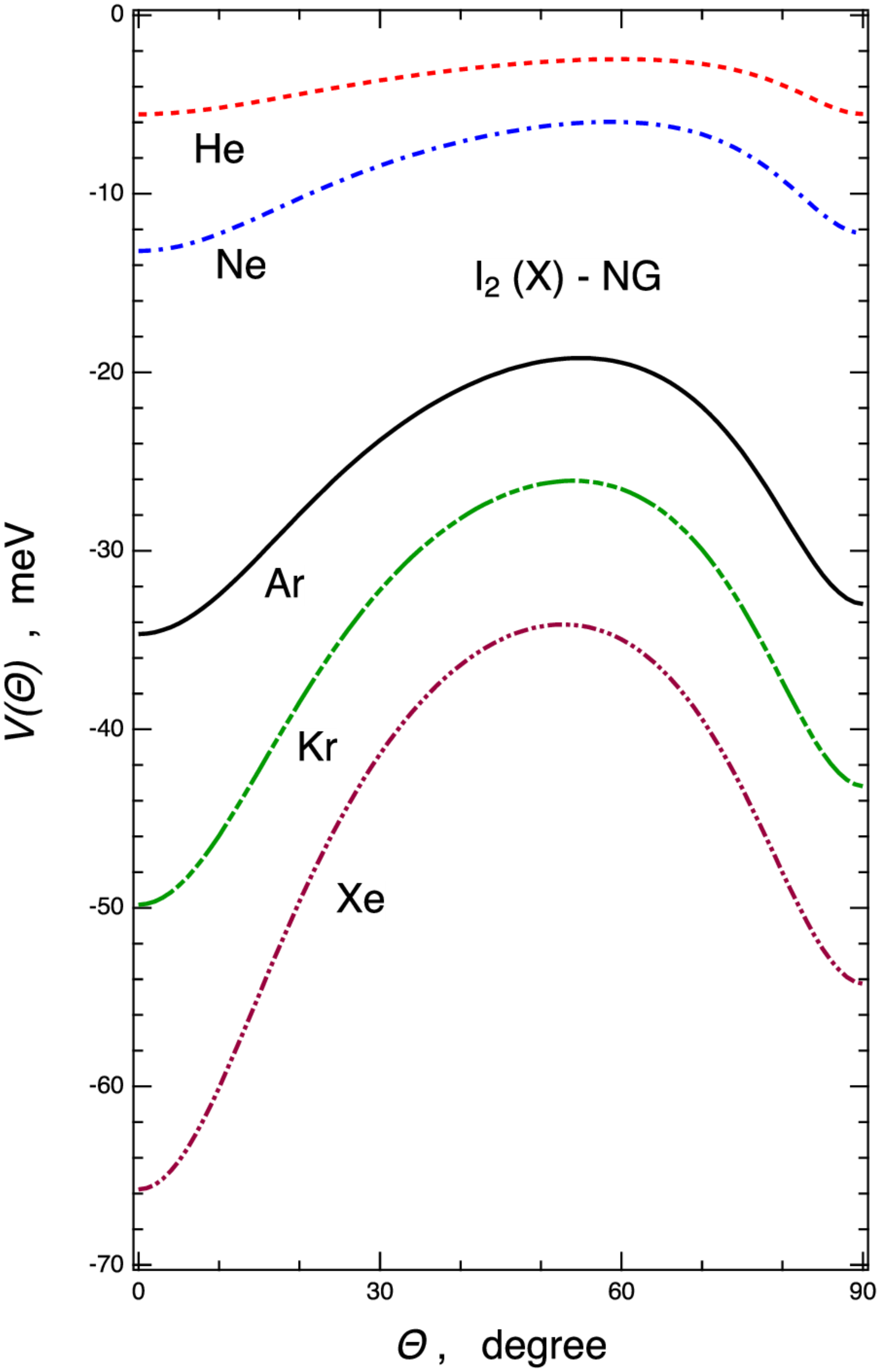

Figure S4. Angular MEP for the Ng-I_2 complexes in the ground *X* state of I_2, reporting the interaction energy, as derived from the potential parametrization, vs the angular variable Θ.

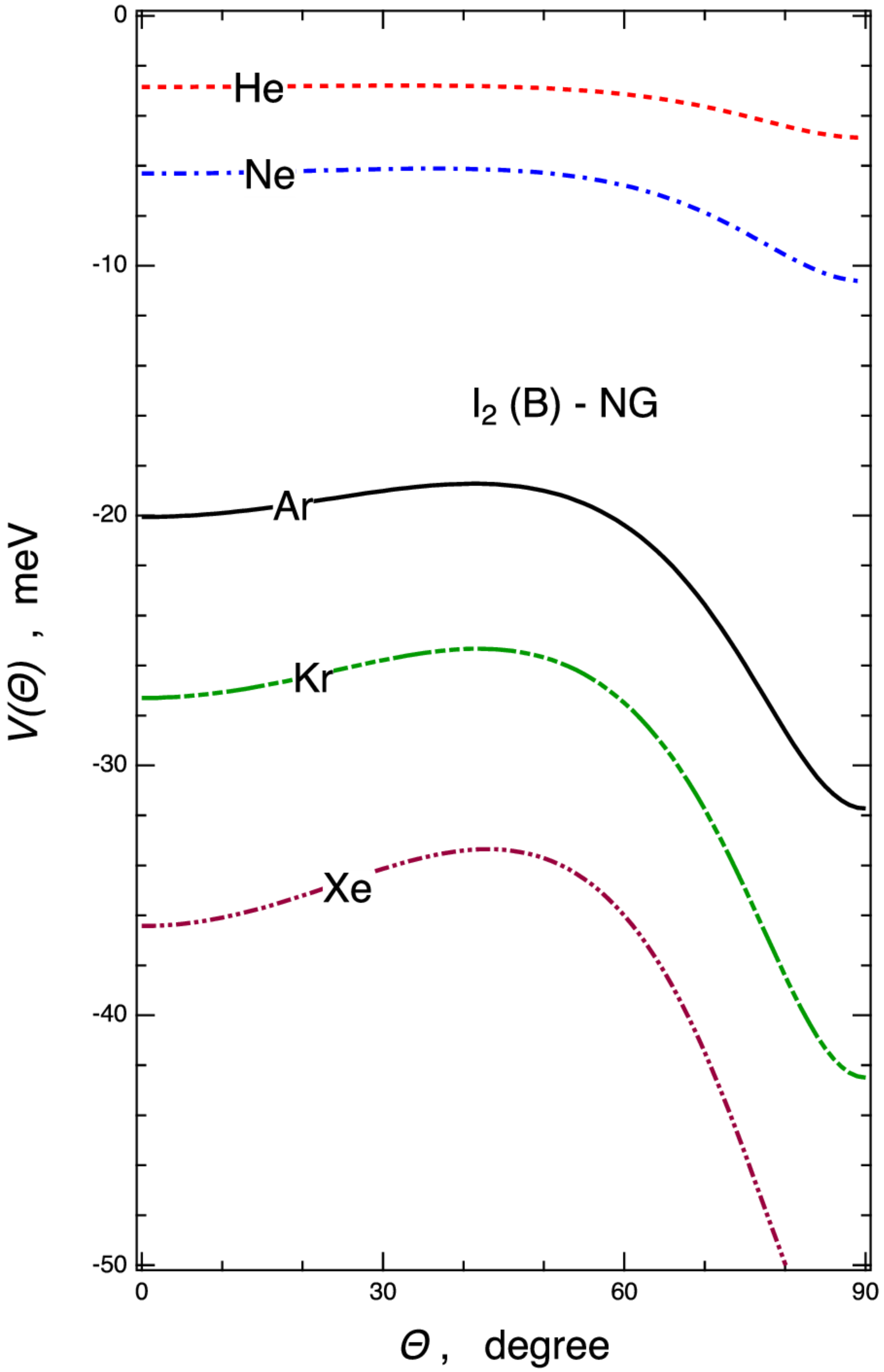

Figure S5. Angular MEP for the Ng-I_2 complexes in the excited *B* state of I_2, reporting the interaction energy, as derived from the potential parametrization, vs the angular variable Θ.

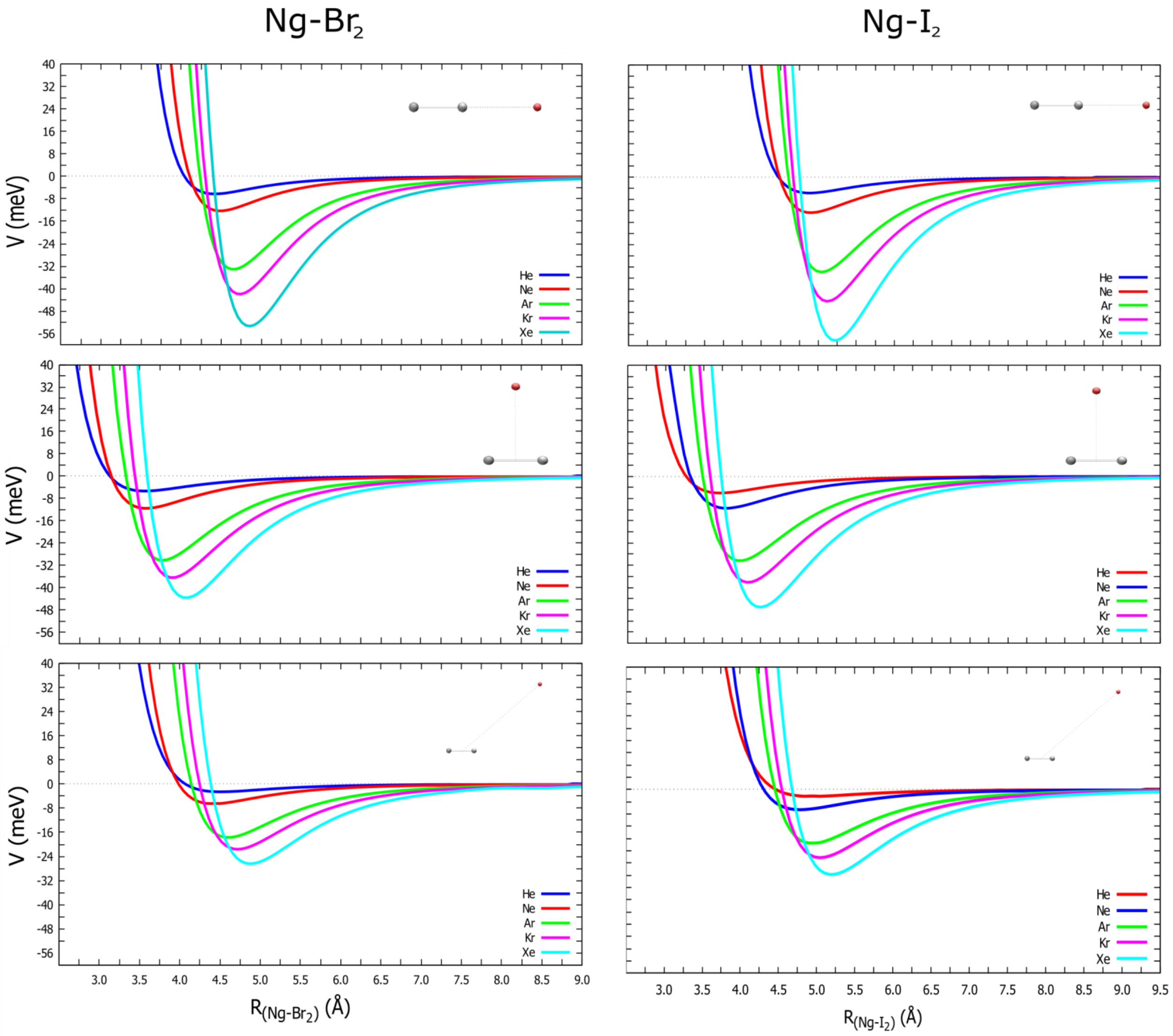

Figure S6. CCSD(T) potential energy curves (interaction potential *V* vs. Ng-Br_2 distance R) for the ground state Ng-Br_2 (AV5Z) and Ng-I_2 (AVQZ) complexes.

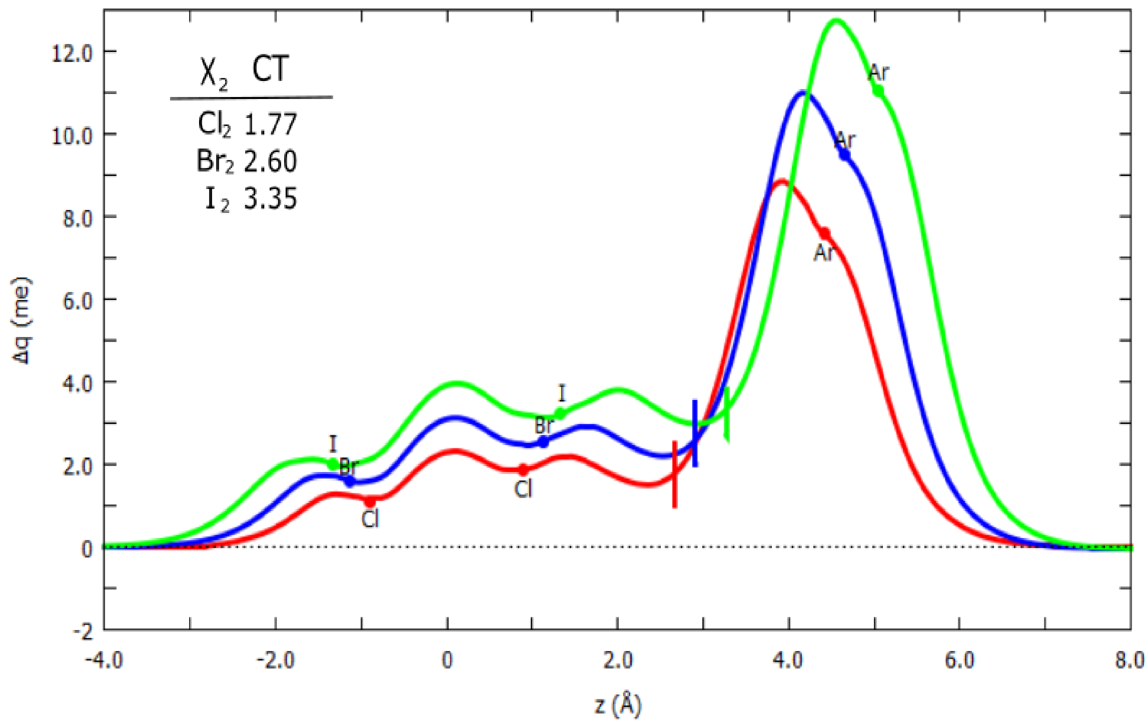

Figure S7. Charge displacement functions (CCSD/AVQZ) of Ar-X_2 ($X\ ^1\Sigma_g^+$) complexes in the linear configuration. Dots on graphs represent the position in the z-axis of the different atoms nuclei. Vertical lines are the isodensity boundary.

Table S1. Equilibrium distances R_m (Å) and binding energy values E_m (meV) for Ng-Br_2 complexes in both ground ($X\,^1\Sigma_g^+$) [CCSD(T)/AVXZ, X = T, Q, 5] and excited ($B\,^3\Pi_u$) [UCCSD(T)/AVTZ] states in the three relative configurations (linear, T-shaped, saddle). In parenthesis the energy values with BSSE corrections.

	Linear		T-Shaped		Saddle (50°)	
**Basis**	R_m	E_m	R_m	E_m	R_m	E_m
			**He-Br$_2$** ($X\,^1\Sigma_g^+$)			
**AVTZ**	4.46	6.73 (4.61)				
**AVQZ**	4.43	6.21 (5.36)	3.57	5.33 (4.70)	4.52	2.58 (2.41)
**AV5Z**	4.42	6.27 (5.67)	3.56	5.35 (5.02)	4.51	2.58 (2.41)
			**Ne-Br$_2$** ($X\,^1\Sigma_g^+$)			
**AVQZ**	4.48	13.21 (10.03)	3.57	12.06 (9.59)	4.41	7.02 (5.35)
**AV5Z**	4.48	12.31 (10.83)	3.57	11.57 (10.49)	4.42	6.56 (5.86)
			**Ar-Br$_2$** ($X\,^1\Sigma_g^+$)			
**AVTZ**	4.71	31.41	3.83	28.75	4.64	17.54
**AVQZ**	4.66	32.35 (28.62)	3.79	29.16 (26.40)	4.62	17.29 (15.25)
**AV5Z**	4.65	33.10 (30.38)	3.78	30.22 (28.07)	4.60	17.71 (16.31)
			**Kr-Br$_2$** ($X\,^1\Sigma_g^+$)			
**AVQZ**	4.75	41.00 (37.18)	3.91	35.59 (32.73)	4.73	21.31 (19.36)
**AV5Z**	4.72	41.66 (39.06)	3.90	36.45 (34.76)	4.72	19.15 (18.40)
			**Xe-Br$_2$** ($X\,^1\Sigma_g^+$)			
**AVQZ**	4.86	52.20	4.09	42.41	4.89	25.86
**AV5Z**	4.85	53.36	4.07	43.67	4.88	26.41
			**Ar-Br$_2$** ($B\,^3\Pi_u$)			
**AVTZ**	5.29	18.80	3.84[(a)]	25.79[(a)]	4.60[(c)]	20.39[(c)]
			3.82[(b)]	27.00[(b)]	4.77[(d)]	16.78[(d)]

[(a)] B_2 symmetry. [(b)] A_1 symmetry.

Table S2. V_{CT} components and CT constant k evaluated at the optimized equilibrium distances (R_{Ng-X_2}, Å).

Ng	R_{Ng-Cl_2}	V_{CT}	k	R_{Ng-Br_2}	V_{CT}	k	R_{Ng-I_2}	V_{CT}	k
He	4.11	1.73	5.00	4.43	2.34	4.84	4.88	2.21	4.02
Ne	4.20	3.66	5.16	4.48	5.34	5.18	4.90	5.77	4.58
Ar	4.40	7.23	5.09	4.66	11.41	4.39	5.04	13.99	4.18
Kr	4.52	12.27	5.00	4.75	17.82	4.73	5.12	22.02	4.45
Xe	4.66	15.14	4.55	4.86	24.91	4.46	5.22	31.71	4.16
